# Dynamic effect of metro-induced vibration on the rammed earth base of the Bell Tower

**DOI:** 10.1186/s40064-016-2627-1

**Published:** 2016-06-30

**Authors:** Jinxing Lai, Fangyuan Niu, Ke Wang, Jianxun Chen, Junling Qiu, Haobo Fan, Zhinan Hu

**Affiliations:** School of Highway, Chang’an University, Xi’an, 710064 China; China Railway First Survey and Design Institute Group Co., Ltd., Xi’an, 710043 Shaanxi China; School of Civil Engineering, Shijiazhuang Tiedao University, Shijiazhuang, 050043 China

**Keywords:** Metro vibration, The Bell Tower, FEM, Tunnel, Peak particle velocity (PPV)

## Abstract

Xi’an Bell Tower (the Bell Tower) is a state-level ancient relic in China. The vibration caused by metro will exert adverse effect on the Bell Tower. This paper aims at presenting 3D-FEM models to predict the peak period velocity (PPV) of rammed earth base when the metro passing through the Bell Tower. The calculation results are compared with those of field test. Both results were found to be in good agreement. Furthermore, the results indicated that the effect of shock absorption measures is significant. The shock absorption tracks can obviously decrease the vibration of the Bell Tower, and the maximum decrease of PPV of the rammed earth base is 78.91 %. The proposed prediction has the potential to be developed as a decision and management tool for the evaluation of the risk associated with the influence of vibration caused by metro on buildings in urban areas.

## Background

As one of four ancient capitals in the world, Xi’an was once the capital for 13 dynasties in Chinese history. The Bell Tower, as a city landmark, is a precious ancient building with a history of over 600 years, but its durability has been gradually weakened for the long history. Xian is now enjoying a boom in metro construction, which will unavoidably cause vibration for the Bell Tower. In the other word, even the tiniest vibrations may lead to fatigue failure for the Bell Tower since the continuous vibration.

In 1970s to 1990s, some researches analyzed the deformation and failure rules of the ancient structures under the vibration according to field tests (Mata 1971; Rueker 1982; Ellis 1987; Clemente and Rinaldis 1998). At the same tine, some researches on prevention of ancient buildings against transportation vibration are made by some European and American experts (Lang 1971; Dawn and Stanworth 1979; Kurzweil 1979). In addition, some new methods, such as artificial neural networks and numerical simulations, are used to address the vibration features of buildings under seismic wave (Degrande and De Roeck 1998; Degrande and Lombaert 2001; Lombaert et al. 2006; Real et al. 2015; Real 2014; Lai et al. 2014, 2015a, 2016b; Ye et al. 2014; Li et al. 2013; Han and Jia 2015; Han et al. 2014). In recent years, some scholars studied the structural and mechanical characteristics under the vibration energy. For example, Dr. Jia Yingxun et al. studied the influence of vibration of Beijing Metro Lines 6 and 8 on ancient buildings (Luo et al. 2015; Yu and Fang 2006; Jia et al. 2009; Xie 2008). Research results indicated that the dynamic response of ancient building structure caused by train vibration changed along with the change of horizontal and vertical distances. On the other hand, to better understand and preserve ancient ruins against train-induced vibrations, vibration measurements and FE analysis were conducted on the Hangu Pass, Luoyang, China, located adjacent to the Longhai railway line (Ye et al. 2015), and the results showed that the set isolation trench can protect the ancient ruin against environmental vibration. In order to ensure the safety and stability of subway tunnel in the practical operation of demolition blasting of the viaduct, Zhao et al. (2015) put forward composite protective structures of steel-rubber tires and makes safety checking calculation of the subway tunnel on the basis of composite protective measures by numerical simulation, and the composite protection system was further optimized.

The influence of metro vibration on REB of the Bell Tower was studied in this paper which will provide valuable experience for protecting similar ancient cultural relics. In order to make an evaluation on the stability of the Bell tower, the field investigation was conducted in the paper firstly that can provide soil parameters for finite element model of the Bell Tower and get the soil layer structure and engineering characteristics. What is more, here we summarized a lot of vibration safety standards of buildings, and got the most suitable vibration safety standards for the Bell Tower. Thirdly, 3D-FEM models are made to simulate the working conditions including with or without shock absorption tracks and different train speeds. Finally, the calculation results are compared with field tests for verifying the correctness in the paper.

## Overview of the Bell Tower

**Fig. 1:**
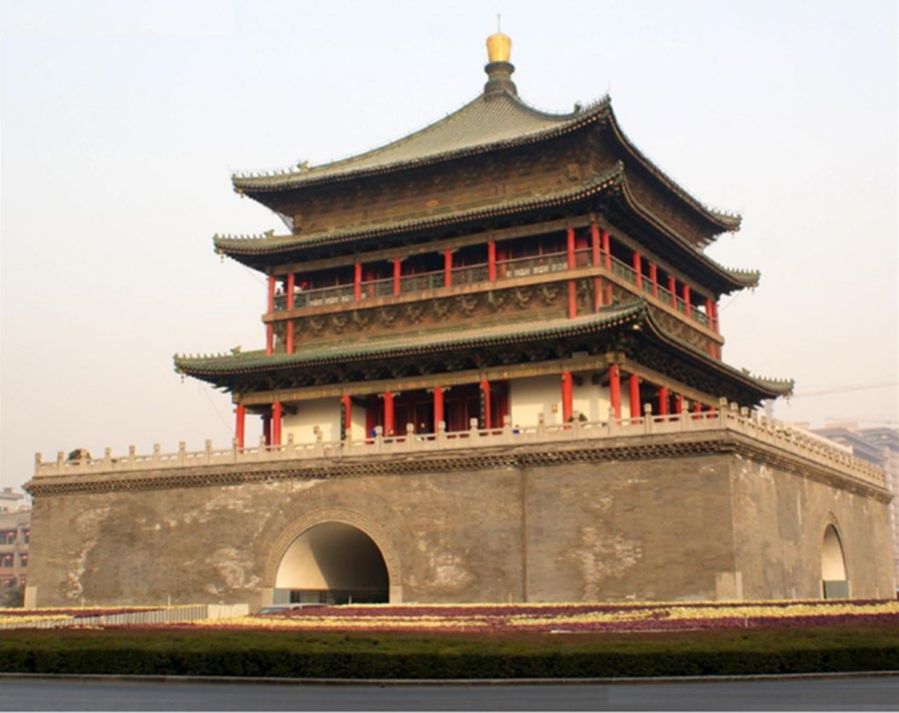
The Bell Tower

**Fig. 2:**
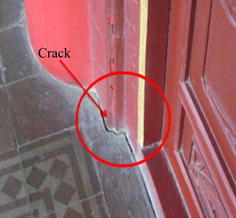
Crack

**Fig. 3:**
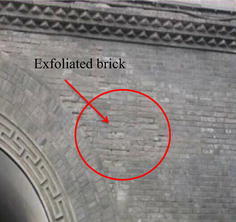
Exfoliated brick

**Fig. 4:**
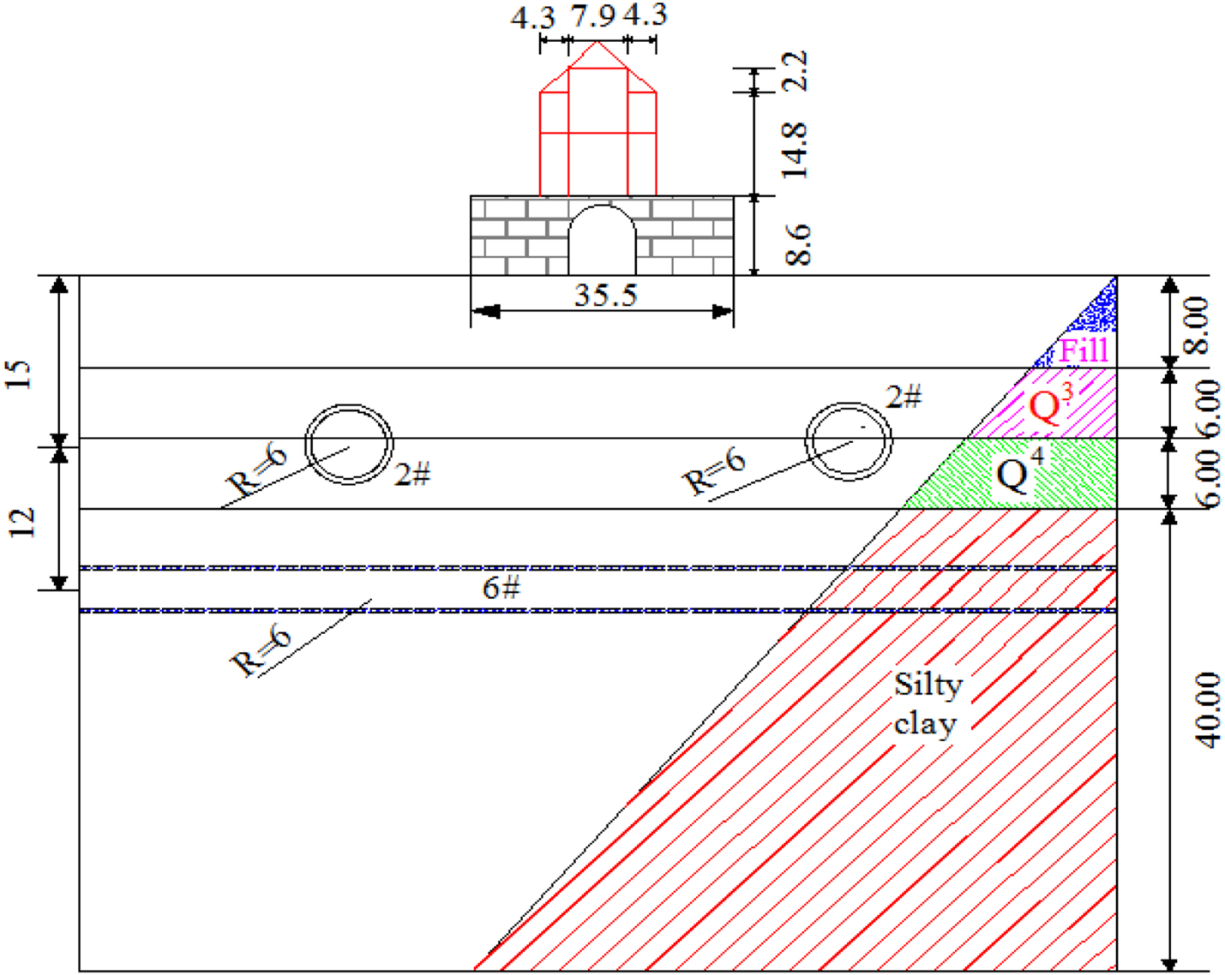
Typical cross-sectional profile soil properties (m)

As the symbol of Xi’an, the Bell tower was built in Ming dynasty, and was a classical brick-and-wood structure. The tower body was borne by 4 central cylinders and 12 surrounding cylinders, as shown in Fig. 1. The cylinders are connected by beams with the large depth-span ratio (Meng 2009). The Bell Tower was facing a lot of damage though many renovations had been done in history. An evaluation work on the Bell Tower was performed in 2008, and damages were observed as shown in Figs. 2 and 3, and the specific geological survey was shown in Fig. 4 (Chen 2008).

## Vibration safety standards

There are different vibration safety standards in different countries, including America, UK, Germany, Japan, Portugal, Swiss, and China, as shown in Table 1. These standards are greatly different for the different environment and structure. However, the physical parameters for analyzing the influence of vibration on buildings are always vibration speed and frequency which are directly related to the damage degree of structures, and playing a decisive role. The standard “Xi’an City Scheme on Protection of Cultural Relics When Urban Track Rapid Transit Line 2 Goes through Bell Tower and City Wall”, formulated by State Administration of Cultural Heritage, was validated by comparing these vibration standards, namely “the PPV of the Bell Tower and City Wall caused by metro vibration is suggested to be controlled within 0.15–0.2 mm/s” (Ma et al. 2016).

**Table 1:** Summary of vibration guide values for structure damage

Vibration standard	Evaluating index	Evaluation object	Evaluation value (mm/s)
ISO 4866:2010 (ISO 2010)	PPV	Ancient architecture	2.5
Germany DIN4150-3:1999 (Germany 1999)	PPV	Ancient architecture	3–10
UK BS7385-2 (UK 1993)	PPV	Ancient architecture	7.5
Switzerland 6.10SN640312:1992 (Swiss 1992)	PPV	Old and poorly maintained buildings	2
Portugal (Meng 2009)	PPV	Ancient architecture	2.5
Japan (Cao 2006)	PPV	Ancient architecture	5
China GB10070-88 (China 2003)	PPV	Ancient architecture (cracking and weathering)	1.8–3.0
China GB/T50452:2008 (China 2008)	PPV	Ancient architecture	3–5
State Administration of Cultural Heritage (Meng 2009)	PPV	The Bell Tower and the City Wall	0.15–0.2

## Numerical analysis

### Research content

There are two metro lines under the Bell Tower, Metro Lines 2 and 6 (2# and 6#, Fig. 4). There were six combinations of the Metro Lines in the finite element model (Table 2), and the train speed was 20, 40, 60 and 80 km/s for each combination.

**Table 2:** Six combinations

Condition	Track type	Metro combination	Train amount
1	Conventional track	2# + 6#	4
2	Conventional track	2# + 2#	2
3	Conventional track	6# + 6#	2
4	Shock absorption track	2# + 6#	4
5	Shock absorption track	2# + 2#	2
6	Shock absorption track	6# + 6#	2

### Numerical model

**Fig. 5:**
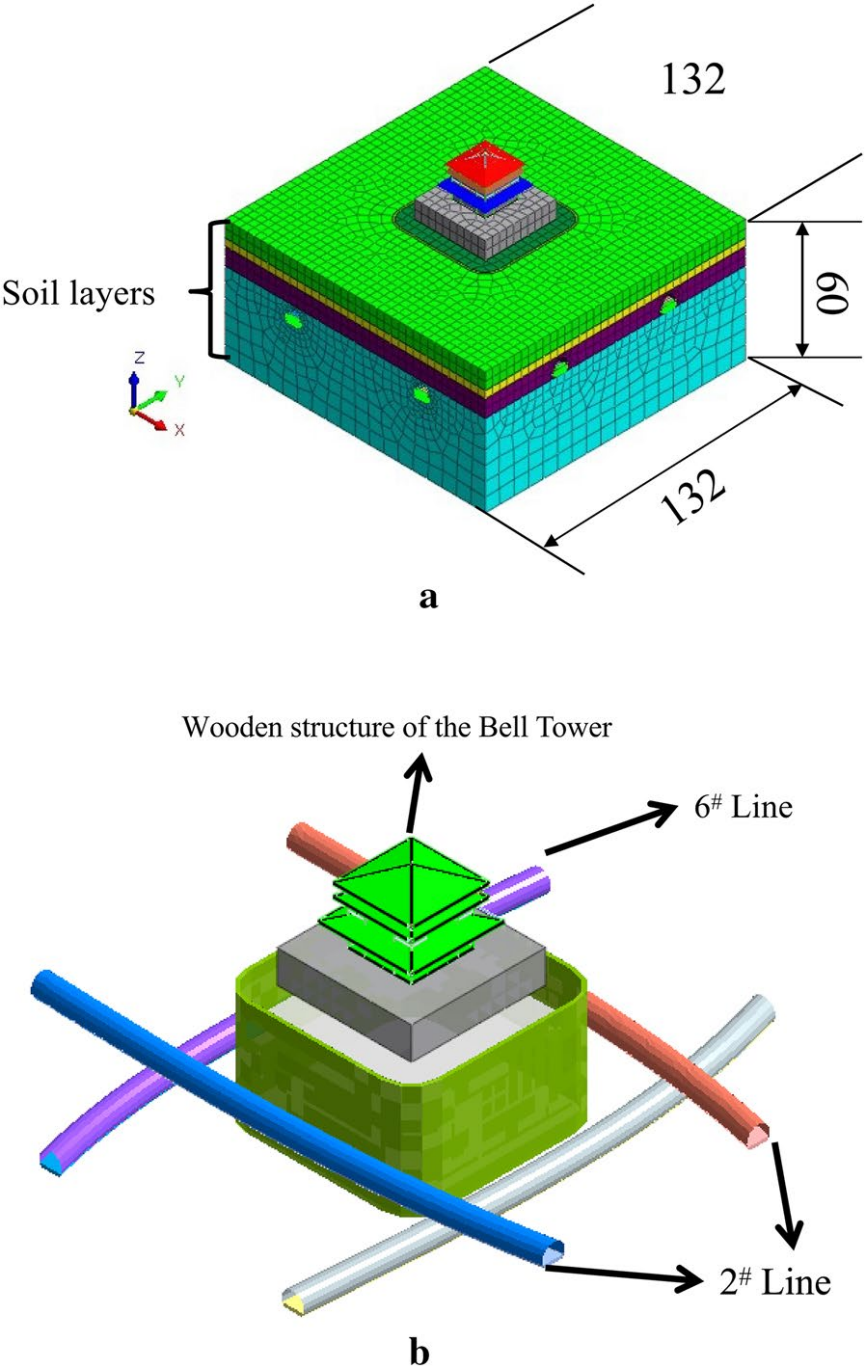
Mesh model (m). **a** Model size, **b** Tunnel location

In this study, 3D-FEM models to predict the influence of Metro Lines 2 and 6 on the Bell Tower were presented by MIDAS/NX (MIDAS Co., Ltd) (Fig. 5). The conventional, masonry structure, soil layers and shock absorption tracks were defined as solid element, tunnel lining was defined as shell element, jet grouting piles under the masonry structure were defined as plate element, wooden beams and columns were defined as beam element, and steel springs on floating slab of shock absorption tracks were defined as elastic connection element (whose rigidity are 6900 kN/m, spacing is 1.2 m) (Meng 2009). The model soil was simplified to four layers, and the model soil parameters were shown in Table 3 (Chen 2008), the other model parameters were shown in Table 4.

**Table 3:** Numerical model soil parameters

Parameter	Unit	Fill	Q^3^	Q^4^	Silty clay
Height (*H*)	(m)	8	3.5	8	40
Soil Young modulus (*E*)	(MPa)	110	403	495	622
Poisson’s ratio (*ν*)	(–)	0.175	0.163	0.160	0.158
Cohesion (*C*)	(kPa)	24.3	25.3	37.5	35.5
Angle of internal friction (*φ*)	(°)	12	12.5	20.2	25.2
Soil unit weight (*γ*)	(kg/m^3^)	17.8	20.4	20.2	20.6

**Table 4:** Unit parameters

Unit	*ρ* (g/cm^3^)	*E* (Gpa)	*ν*	*C* (Kpa)	*Φ* (*°*)
Jet grouting piles	2.3	39	0.29	–	–
REB	1.67	3	0.3	48	35
Tunnel lining	2.5	40	0.29	–	–
Track	2.4	46	0.3	–	–

### The time period and time increment size

According to different sampling frequency, the time period and time increment size of the environmental vibration problem are studied in many Literatures (Ma et al. 2016; Lai et al. 2016a, b, c). The research results show that when the minimal time period of the model is 50 times as much as the time increment size, the error of the calculation results can not be considered. The ground time-period of the Bell Tower is 0.29–0.4 s, so when the time increment size is 0.005 s, the computational accuracy meets the requirements.

### Dynamic analysis equation

The motion equation of the structural system based on Hamilton principle (Meng 2009) is:

1 $$[M]\left\{ {{\ddot{u}}} \right\} + [C]\left\{ {\dot{\ddot{{u}}}} \right\} + [K]\left\{ u \right\} = \left\{ {F(t)} \right\}$$

where [*M*], [*C*] and [*K*] are the mass matrix, damping matrix, and stiffness matrix of the system, respectively, and they are combined with the mass matrix, damping matrix, and stiffness matrix of all units together; $\{{\ddot{u}}\} ,\,\{ \dot{u}\} ,$ and {*u*} are the acceleration vector, velocity vector and displacement vector of the system, respectively; *F*(*t*) refers to nodal force vector changing along with time. The mass matrix, damping matrix and stiffness matrix as well as nodal force vector are calculated as follows:

2a $$[M_{e} ] = \iiint {\rho [N]^{T} [N]dV}$$

2b $$[C_{e} ] = \iiint {c[N]^{T} [N]dV}$$

2c $$[K_{e} ] = \iiint {[B]^{T} [D][B]dV}$$

2d $$[F_{e} ] = \iiint {[N]^{T} \left\{ f \right\}dV} + \iint {[N]^{T} [\bar{P}]}dB$$

Generally, it is assumed that the relationship between the damping matrix and mass matrix, stiffness matrix is in direct proportion, that is Rayleigh damping is adopted (Chen 2008), and the expression is as follow:

3 $$\left[ C \right] = \alpha \left[ M \right] + \beta \left[ K \right]$$

According to the orthogonal condition of the vibration mode, the undetermined constants *α* and *β* and the damping ratio should satisfy the following relationship:

4 $$\xi_{k} = \frac{\alpha }{{2\omega_{k} }} + \frac{{\beta \omega_{k} }}{2}\quad (k = 1,2, \ldots ,n)$$

where *ξ*_*k*_ is the damping ratio; *ω*_*k*_ is the inherent frequency; *α* and *β* are damping ratio coefficients.

The free vibration equation of the system can be used to calculate the inherent frequency of *ω*_*i*_ and *ω*_*j*_, and based on tests and similar structure data, the damping ratios of *ξ*_*i*_ and *ξ*_*j*_ can be obtained. From formula (4), *α* and *β* are obtained. If *ξ*_*i*_ = *ξ*_*j*_, *ω*_0_ and *ξ*_0_ is the fundamental frequency of system and the damping ratio of corresponding vibration mode, respectively, then the following relational expression can be obtained:

5 $$\left\{ {\begin{array}{*{20}l} {\alpha = \xi_{0} \omega_{0} } \hfill \\ {\beta = \xi_{0} /\omega_{0} } \hfill \\ \end{array} } \right.$$

### Boundary conditions

When finite element method is adopted to simulate the vibration, the truncation boundary will generate reflection, which will result in distortion of calculation. To avoid distortion, spring viscous artificial boundary is set in this model (Meng 2009). Viscous artificial boundary not only can simulate the excluded soil and its rigidity in finite element model, but also can avoid the reflection of truncated boundary (MIDAS Co., Ltd). The lecture (Chen 2008) suggests the artificial viscous damping force in two directions along boundary surface, with the magnitude:

6 $$\left\{ {\begin{array}{*{20}l} {\bar{\sigma } = a\rho v_{\text{P}} \dot{w}} \hfill \\ {\bar{\tau } = b\rho v_{\text{S}} \dot{u}} \hfill \\ \end{array} } \right.$$

where $\bar{\sigma }$ and $\bar{\tau }$ are the normal stress and shear stress along artificial boundary, respectively; $\dot{w}$ and $\dot{u}$ are the velocity component in normal and tangential directions along the boundary, respectively; *v*_*P*_ and *v*_*S*_ are the velocity of incident pressure wave and shear wave, respectively; and the values of *a* and *b* can be found in the lecture [28].

### Train load

**Fig. 6:**
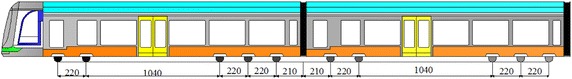
Two carriages and wheel span (cm)

**Fig. 7:**
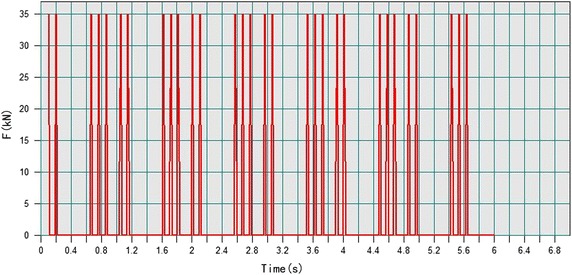
Dynamic load of trains (80 km/h from MIDAS-NX)

The vibration during metro operation is caused by train dynamic load. There are many kinds of trains in MIDAS/NX and it just needs some relevant parameters (MIDAS Co., Ltd), including train type, number of wheels, wheel distance, train speed, running distance, etc. The train formation of Xi’an Metro is six trains (Meng 2009). So the standard train EL-18 was adopted in the model (MIDAS Co., Ltd), and the number and separation distance of wheels were shown in Fig. 6. The vibration response of the Bell Tower was simulated at the speed of 20, 40, 60 and 80 km/h, respectively. The distance that trains pass through the Bell Tower was 60 m. The dynamic load generated at the speed of 80 km/h was shown in Fig. 7.

### Numerical results

A number of numerical simulations have been carried out to investigate the effects of metro vibration to the Bell Tower: (1) the distribution of PPV in REB; (2) the laws of P PV in different condition; (3) the effect of the shock absorption tracks.

#### The PPV of the REB

**Fig. 8:**
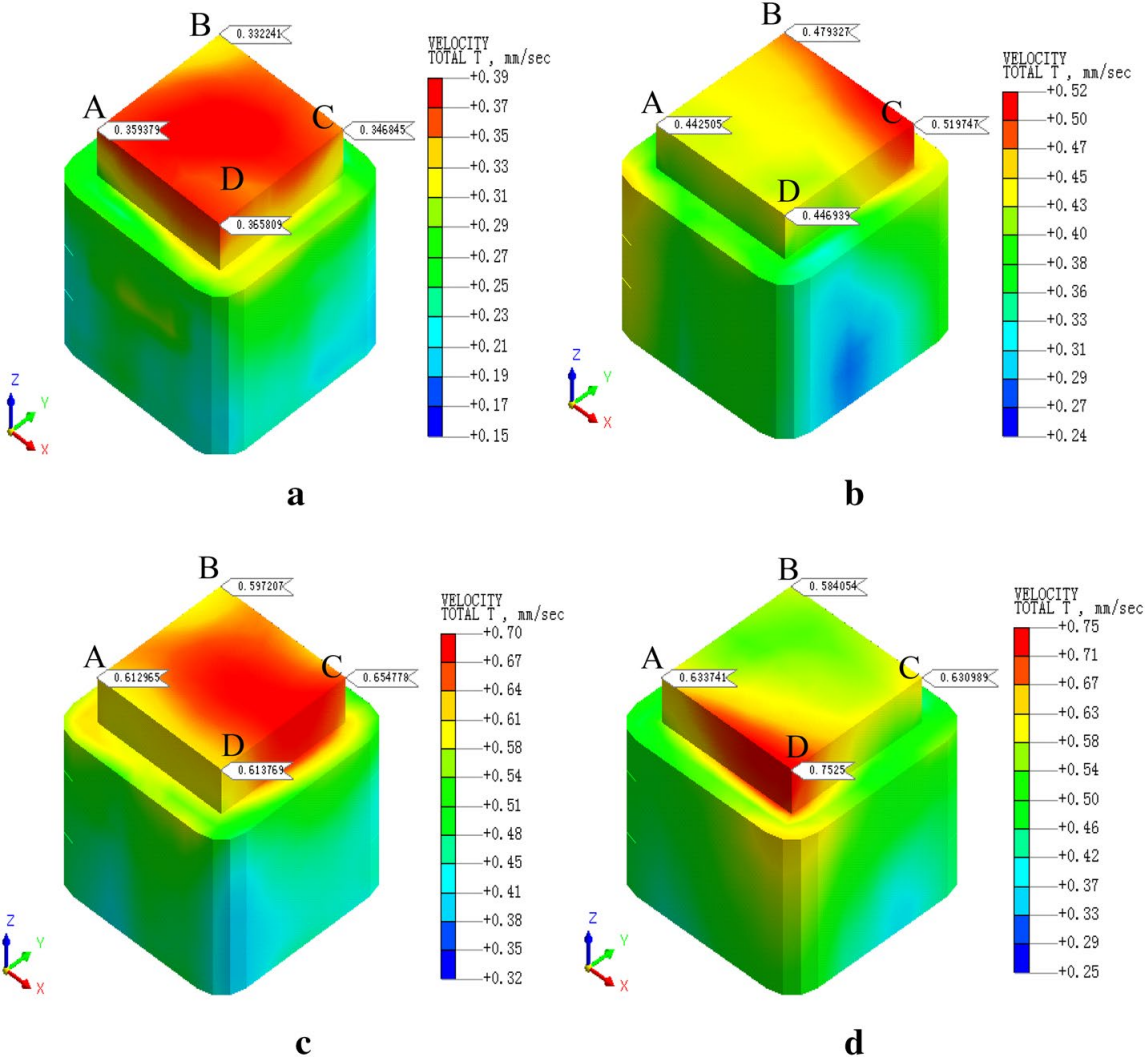
PPV of the condition 1. **a** *v* = 20 km/h, **b** *v* = 40 km/h, **c** *v* = 60 km/h, **d** *v* = 80 km/h

**Fig. 9:**
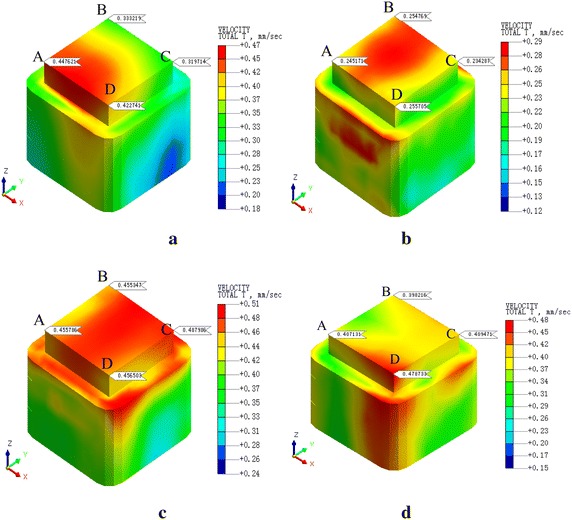
PPV of the condition 2. **a** *v* = 20 km/h, **b** *v* = 40 km/h, **c** *v* = 60 km/h, **d** *v* = 80 km/h

**Fig. 10:**
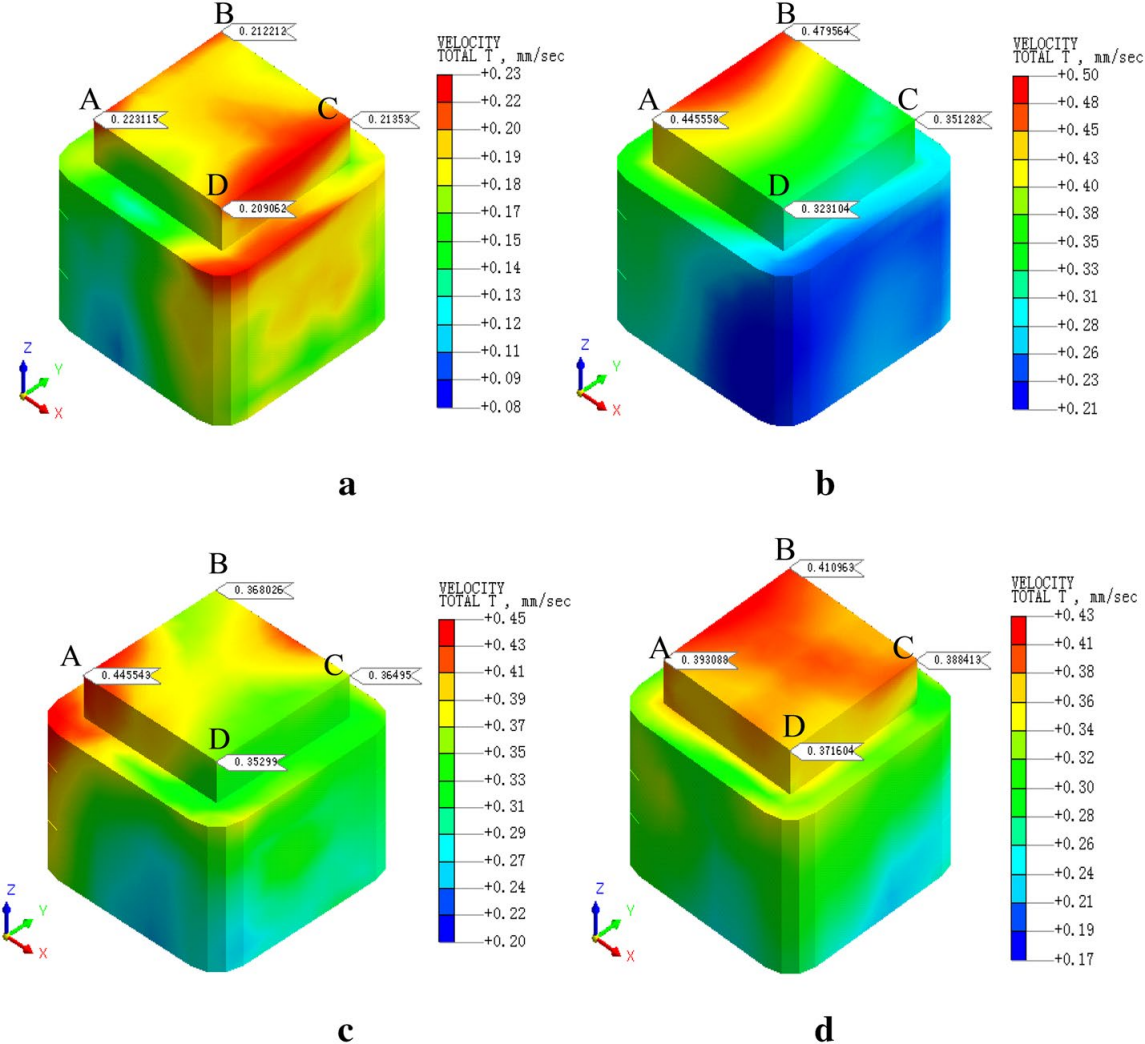
PPV of the condition 3. **a** *v* = 20 km/h, **b** *v* = 40 km/h, **c** *v* = 60 km/h, **d** *v* = 80 km/h

**Fig. 11:**
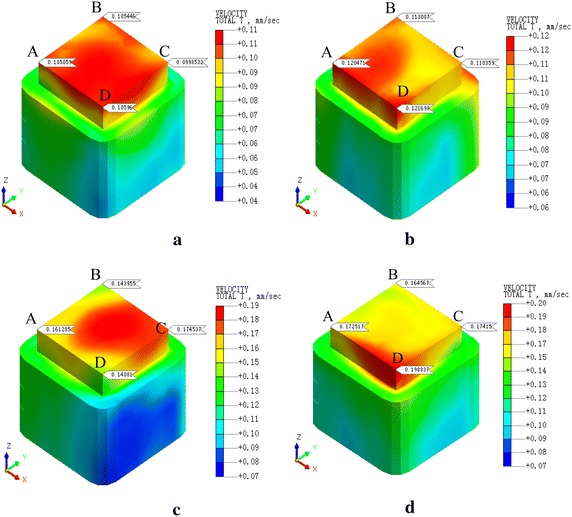
PPV of the condition 4. **a** *v* = 20 km/h, **b** *v* = 40 km/h, **c** *v* = 60 km/h, **d** *v* = 80 km/h

**Fig. 12:**
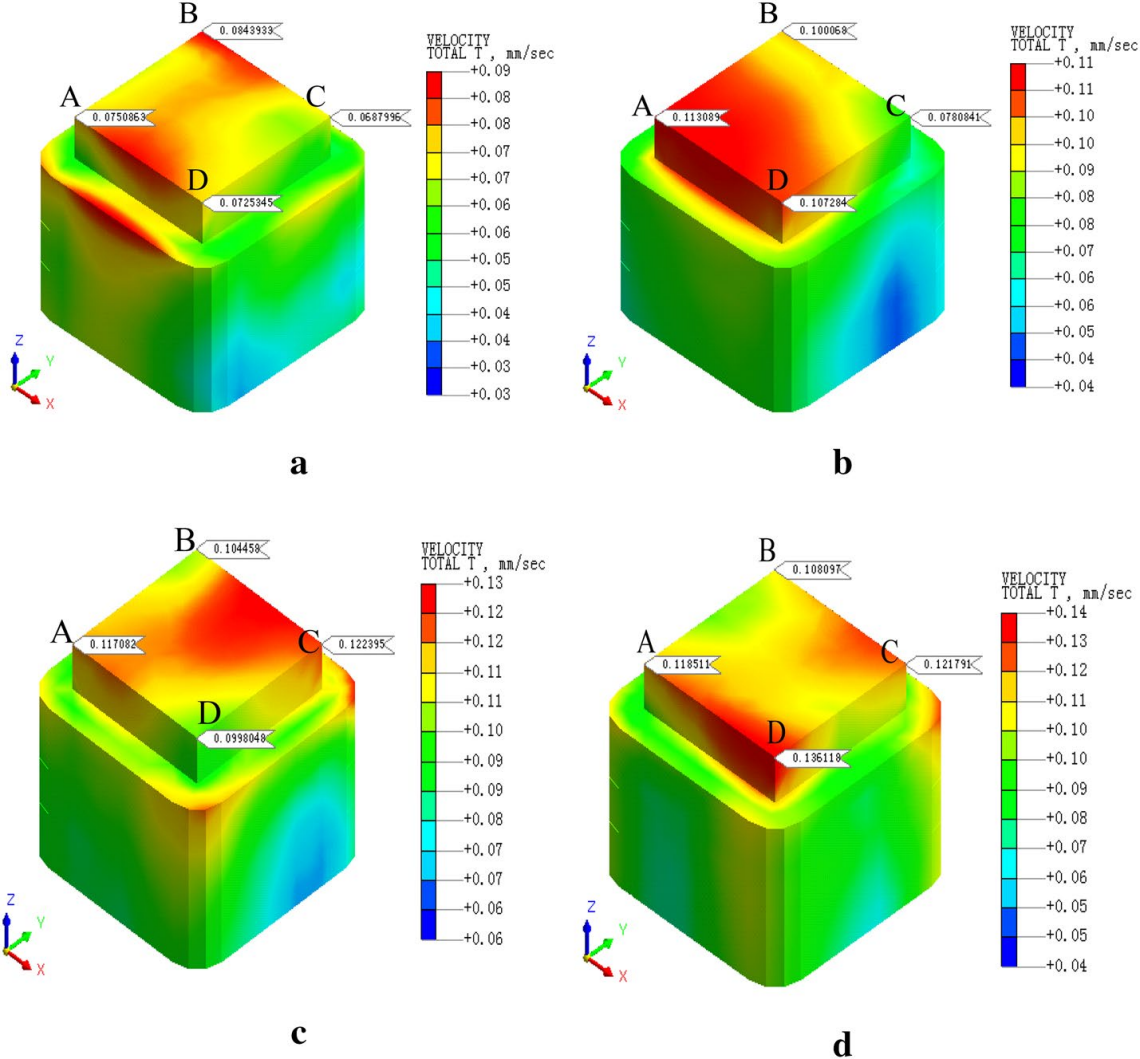
PPV of the condition 5. **a** *v* = 20 km/h, **b** *v* = 40 km/h, **c** *v* = 60 km/h, **d** *v* = 80 km/h

**Fig. 13:**
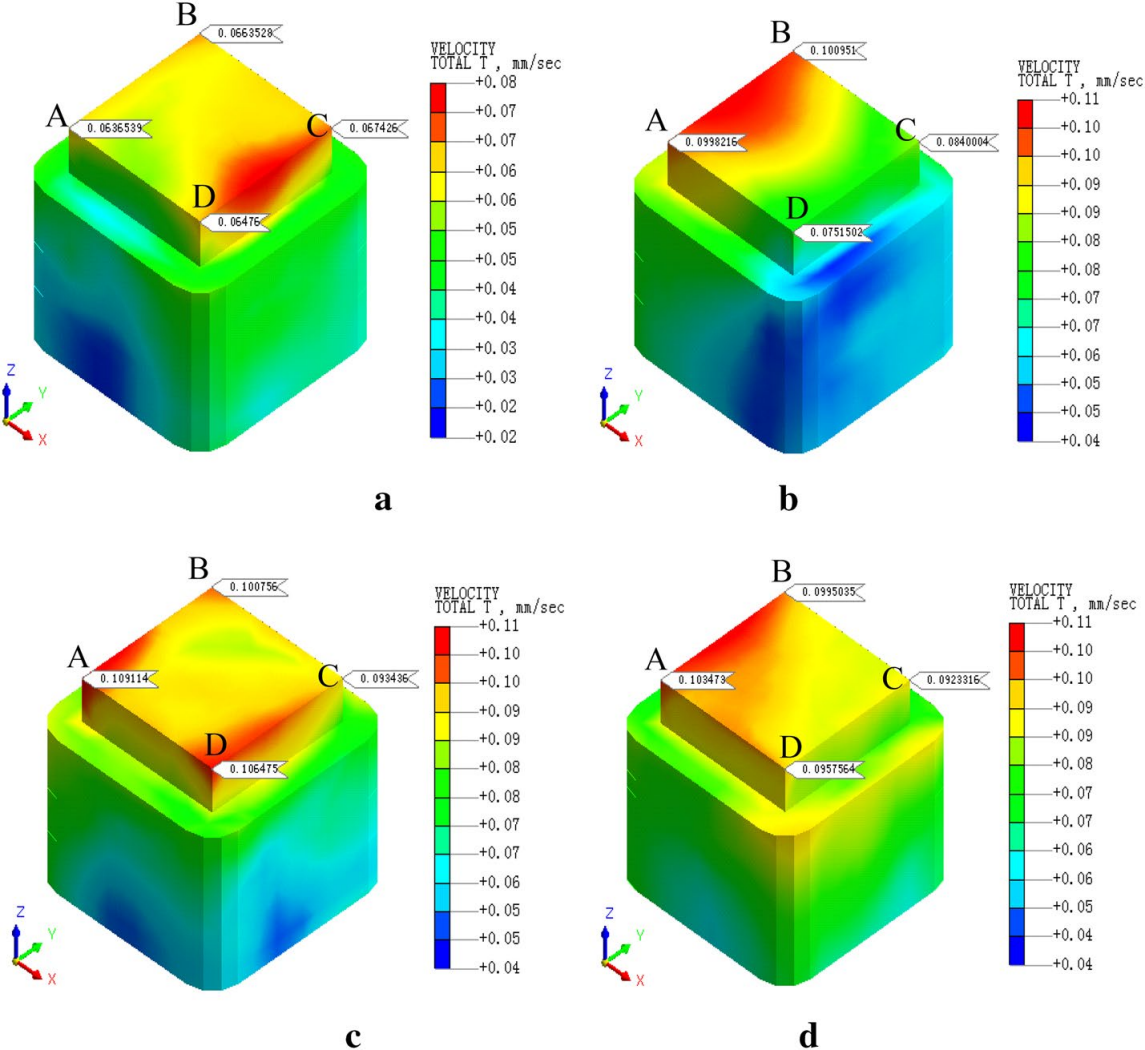
PPV of the condition 6. **a** *v* = 20 km/h, **b** *v* = 40 km/h, **c** *v* = 60 km/h, **d** *v* = 80 km/h

Vibration velocities of the REB in each condition were shown at Figs. 8, 9, 10, 11, 12 and 13. In order to facilitate the analysis, we chose a representative of the four corners of the REB, that is A, B, C, and D. As we can see, the PPV of the corners in each condition changes with the change of train velocity. To the condition 1 and condition 4, when there are four trains, the maximum PPV is corner D when the train velocity is 20 km/h, the maximum PPV is corner C when the train velocity is 60 km/h, and the maximum PPV is corner D when the train velocity is 80 km/h. The only difference is when the train velocity is 40 km/h, where the maximum PPV is corner C in condition 1 but the maximum PPV is corner D in condition 4. To the other four conditions, there are similar rules, as shown in Table 7. The whole results indicated the PPV of four trains is larger than two trains (Table 5), and the location of the maximum PPV is basically consistent when the train number and velocity are identical. What is more, the maximum PPV of condition 1–3 were beyond standard and the condition 4–6 met standard requirements (Table 6).

**Table 5:** PPV of each condition

Condition	1	2	3	4	5	6
PPV	0.752	0.488	0.479	0.199	0.136	0.109
Train velocity	80	60	40	80	80	60
The max location	D	C	B	D	D	A
Figures	Figure 8d	Figure 9c	Figure 10b	Figure 11d	Figure 12d	Figure 13c

**Table 6:** PPV of each condition

Condition	1	4	2	5	3	6
20	D	D	A	A	A	C
40	C	D	D	A	B	B
60	C	C	C	C	A	A
80	D	D	D	D	B	A

#### Distribution laws of PPV

**Fig. 14:**
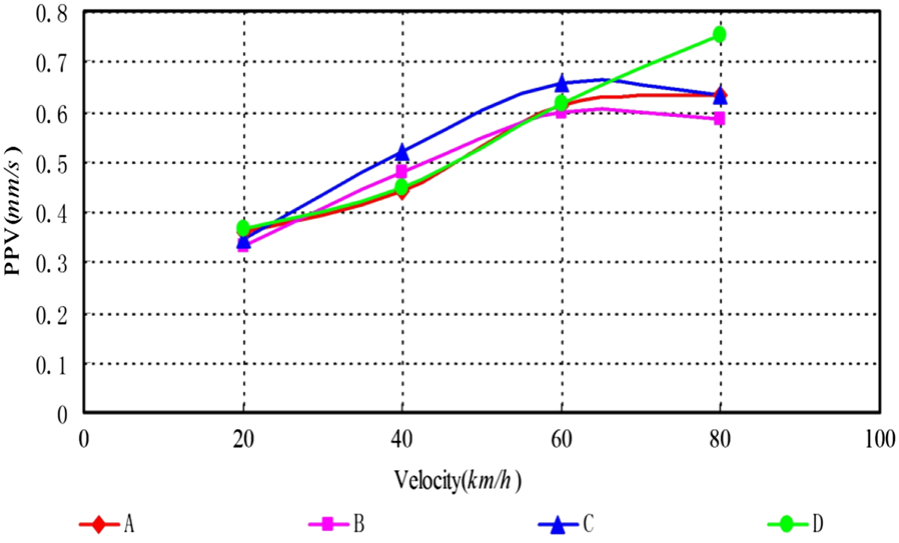
Condition 1

**Fig. 15:**
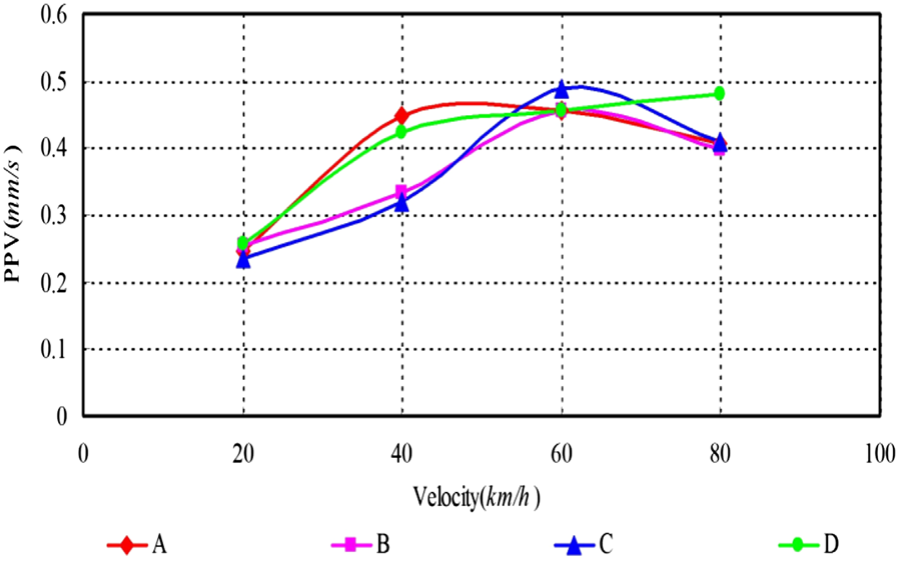
Condition 2

**Fig. 16:**
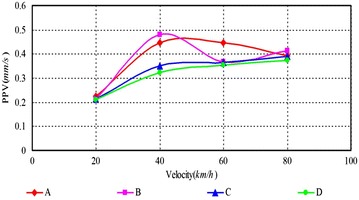
Condition 3

**Fig. 17:**
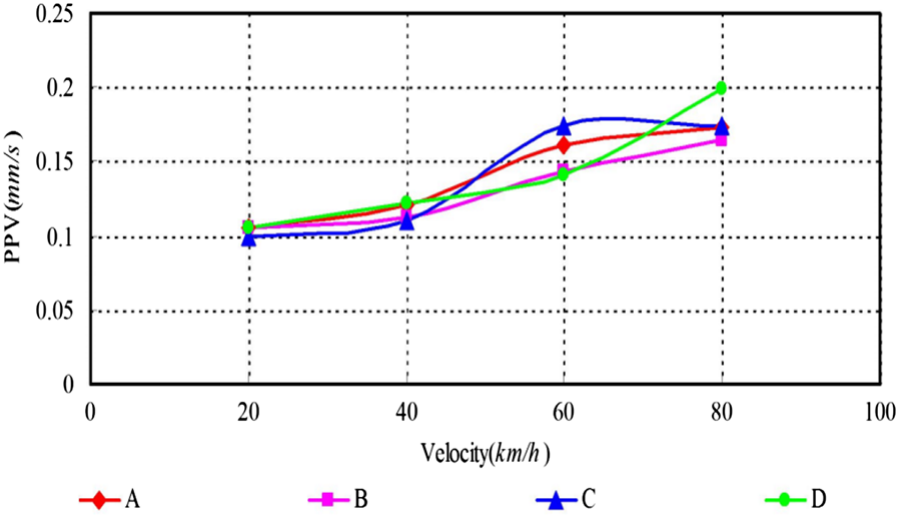
Condition 4

**Fig. 18:**
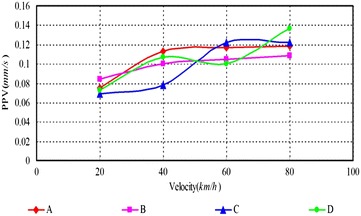
Condition 5

**Fig. 19:**
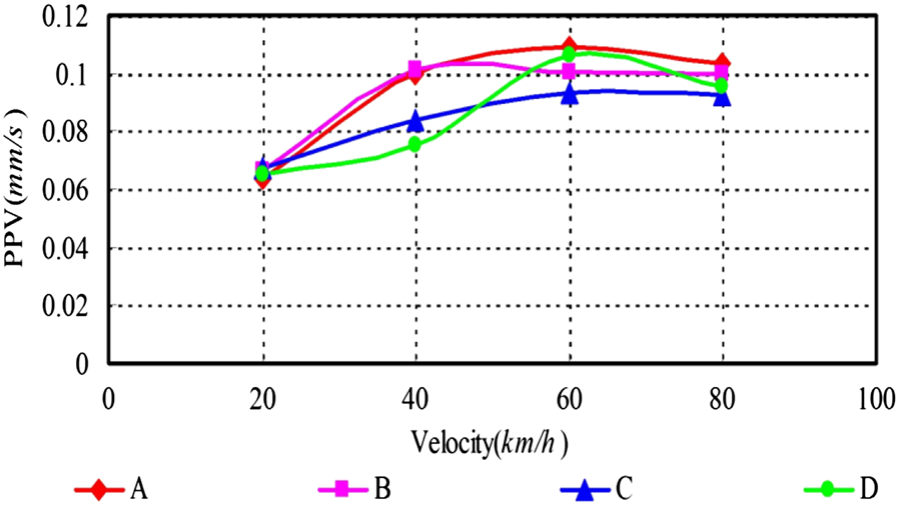
Condition 6

As can be seen, (1) According to Figs. 14, 15, 16, 17, 18 and 19, most of the curve showed a rising trend, but some PPV curve decrease as the velocity increase which is consistent with the regularities obtained in previous studies (Zhao et al. 2015). The reasons are that there are many factors will make effect on the vibration frequency the REB, such as track category, train quantity and the running mode of train. (2) When there were no shock absorption tracks, the PPV of the REB beyond standard, and under the conditions 4–6 (with shock absorption tracks), the PPV was less than the allowable maximum value, which indicated that the vibration velocity of the REB was related to the track category. (3) When condition 1 (C1) versus condition 2 (C2), or C1 versus C3, or C4 versus C5, or C4 versus C6, the PPV of four points (A, B, C and D) in four trains were large than in two trains, which indicated that the vibration velocity of the REB increase with the increase of the train quantity. (4) Comparing the condition 2 with condition 3 or condition 5 with condition 6 (Figs. 15 vs. 16, Figs. 18 vs. 19), when two trains of different routes passing through the Bell Tower at the same time, distribution laws of PPV was different with each other, which indicated that vibration velocity of the REB was related to the running mode of train.

### Effect of the shock absorption measures

To evaluate the effect of the shock absorption tracks much directly, reduction of PPV was shown in Tables 7, 8 and 9.

**Table 7:** Condition 1 versus condition 4

Point	A (mm/s)	B (mm/s)	C (mm/s)	D (mm/s)
Condition 1	0.634	0.584	0.655	0.753
Condition 4	0.172	0.165	0.175	0.199
R (%)	72.87	71.75	73.28	73.57

**Table 8:** Condition 2 versus condition 5

Point	A (mm/s)	B (mm/s)	C (mm/s)	D (mm/s)
Condition 2	0.455	0.455	0.488	0.479
Condition 5	0.117	0.108	0.122	0.136
R (%)	74.29	76.92	75	71.61

**Table 9:** Condition 3 versus condition 6

Point	A (mm/s)	B (mm/s)	C (mm/s)	D (mm/s)
Condition 3	0.445	0.479	0.388	0.372
Condition 6	0.109	0.101	0.093	0.106
R (%)	75.51	78.91	76.04	71.51

$R = \frac{{(P_{1} - P_{2} )}}{{P_{1} }}$, where R is reduction, P1 is PPV with shock absorption tracks, and P2 is PPV without shock absorption tracks

When there were no shock absorption measures, the PPV of the REB was 0.372–0.753 mm/s, beyond the standard value of 0.15–0.2 mm/s. In conditions 4–6 with shock absorption tracks, the PPV of the REB was 0.093–0.199 mm/s, which decreased to the allowable value range. When compared with the condition 1, the PPV of four points in condition 4 reduces by 71.75–73.57 %. Compared with condition 2, the PPV of four points in condition 5 reduces by 71.61–76.92 %. Compared with the condition 3, the PPV of four points in condition 6 reduces 71.51–78.91 %. Thus, the application of shock absorption tracks can largely weaken the influence of vibration on the Bell Tower.

## On-site monitoring

**Fig. 20:**
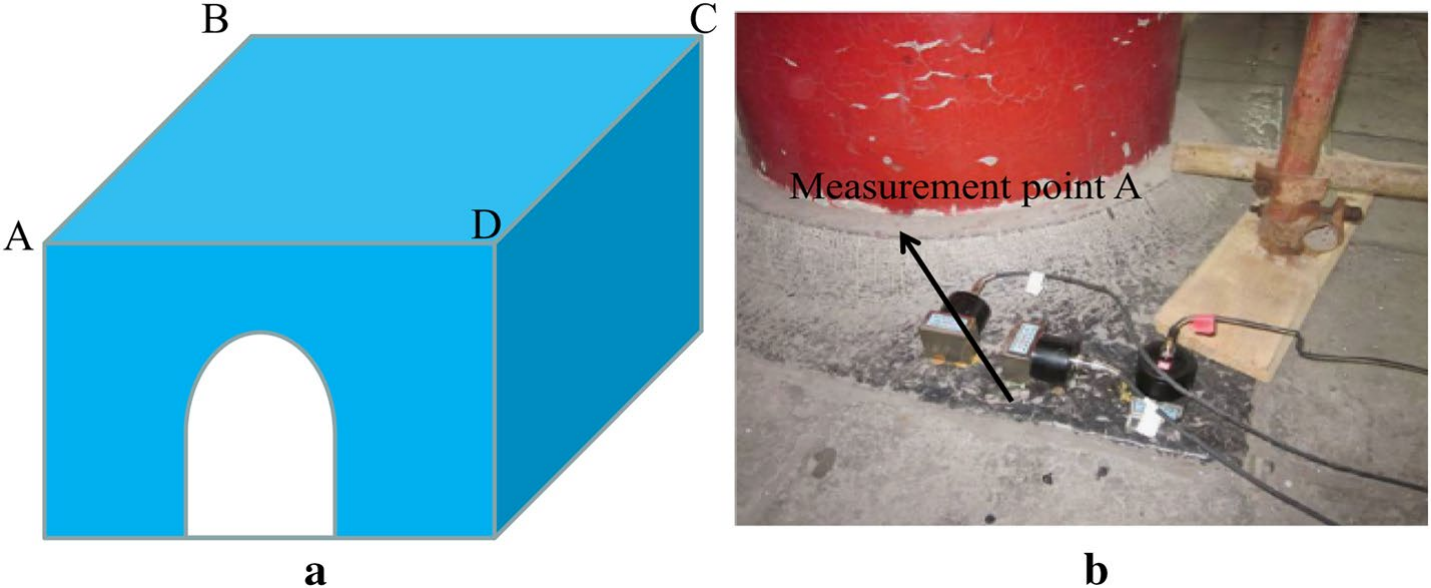
On-site monitoring. **a** Sketch of measurement points, **b** measurement point A (Ma et al. 2016)

To validate the model, calculation results were compared with those of field tests (Ma et al. 2016). As Metro Line 6 has not been finished, this article verifies the vibration response of the Bell Tower in condition 5 when the train velocity is 60 km/h. CMG-40T sensor and REFTEK130-B intelligent signal acquisition device were applied to monitor for a period of 24 h. According to the above analysis, the four points (A, B, C and D) is the key points, so these points were monitored (Fig. 20).

The PPV of four points under in condition 5 were consistent with field data, as shown in Table 10, which indicated that the model in this article can quantitatively reflects the actual vibration response of wooden structure.

**Table 10:** On-site versus calculation results

Point	On-site (mm/s)	Model (mm/s)
A	0.125	0.117
B	0.133	0.108
C	0.148	0.122
D	0.153	0.136

## Conclusions

In order to investigate influence of vibration on ancient architectures, this article summarized vibration standards and took PPV as the safe standard for the Bell Tower. Numerical analyses were done using the MIDAS/NX in six conditions and the following results were observed:

1. The vibration velocity of the REB does not increase monotonically with the increase of the train velocity; however, it is related to the train quantity, track category and running mode of train.
2. When shock absorption tracks are not adopted, the PPV of the REB beyond the safety standard, which has the potential to induce severe damage to the Bell Tower. However, the PPV of the Bell Tower decreases greatly when vibration reduction track is adopted, and the PPV does not exceed the allowable maximum value, and the maximum decreases are 78.91 %. The whole predicted that the application of shock absorption tracks can weaken the influence of vibration on the Bell Tower to a great extent.
3. After the verification on calculation result for the model in this article through on-site monitor, it is discovered that the model can quantitatively reflect the actual vibration response of the Bell Tower. Therefore, we will investigate the settlement of REB and the PPV of the wooden structure caused by vibration for the Bell Tower in the following study.

## References

[CR1] BS7385-2 (1993) Evaluation and measurement for vibration in buildings—part 1. In: Guide for measurement of vibrations and evaluation of their effects on buildings

[CR2] Cao YM (2006). Vibration of high-rise buildings induced by running trains. Eng Mech.

[CR3] Chen RC (2008) Study on effects on Bell Tower due to train-induced vibrations on metro in Xi’an. Ph.D. Thesis, Beijing Jiaotong University

[CR4] Clemente P, Rinaldis D (1998). Protection of a monumental building against traffic induced vibrations. Soil Dyn Earthq Eng.

[CR5] Dawn TM, Stanworth CG (1979). Ground vibration from passing trains. J Sound Vib.

[CR6] Degrande G, De Roeck G (1998). Wave propagation in layered dry, saturated and unsaturated pore elastic media. Solid Struct.

[CR7] Degrande G, Lombaert G (2001). An efficient formulation of Krylov’s prediction model for train induced vibrations based on the dynamic reciprocity theorem. J Acoust Soc Am.

[CR8] DIN4150-3 (1999) Structural vibration Part3. In: Effect of vibration on structures

[CR9] Ellis P (1987). Effect of traffic vibration on historic buildings. Sci Total Environ.

[CR10] GB/50894 (2003). Code for design of environment protection for machinery industry.

[CR11] GB/T50452 (2008). Technical specification for protection of historic buildings against man-made vibration.

[CR12] Han XH, Jia WL (2015). Study on the effect and mechanism of aerodynamic measures for the vortex-induced vibration of separate pairs of box girders in cable-stayed bridges. Shock Vib.

[CR13] Han WS, Yuan SJ, Ma L (2014). Vibration of vehicle-bridge coupling system with measured correlated road surface roughness. Struct Eng Mech.

[CR14] ISO 4866 (2010) Mechanical vibration and shock-vibration of fixed structures-guidelines for the measurement of vibrations and evaluation of their effects on structures

[CR15] Jia YX, Guo M, Liu WN (2009). Dynamic effect of train induced vibration on historic buildings. J Beijing Jiaotong Univ.

[CR16] Kurzweil G (1979). Ground borne noise and vibration from underground rail systems. J Sound Vib.

[CR17] Lai HP, Wang SY, Xie YL (2014). Experimental research on temperature field and structure performance under different lining water contents in road tunnel fire. Tunn Undergr Space Technol.

[CR18] Lai JX, Fan HB, Chen JX et al (2015a) Blasting vibration monitoring of under crossing railway tunnel using wireless sensor network. Int J Distrib Sens Netw, Article ID 703980, 7 pages. doi:10.1155/2015/703980

[CR19] Lai JX, Qiu JL, Chen JX et al (2015b) New technology and experimental study on snow-melting heated pavement system in tunnel portal. Adv Mater Sci Eng, Article ID 706536, 11 pages. doi:10.1155/2015/706536

[CR20] Lai JX, Mao S, Qiu JL et al (2016a) Investigation progresses and applications of fractional derivative model in geotechnical engineering. Math Probl Eng, Article ID 9183296, 15 pages. doi:10.1155/2016/9183296

[CR21] Lai JX, Qiu JL, Feng ZH et al (2016b) Prediction of soil deformation in tunnelling using artificial neural networks. Comput Intell Neurosci, Article ID 6708183, 16 pages. doi:10.1155/2016/6708183 10.1155/2016/6708183 PMC4706869 26819587

[CR22] Lai JX, Wang KY, Qiu JL et al (2016c) Vibration response characteristics of the cross tunnel structure. Shock Vib, Article ID 9524206, 11 pages. http://www.hindawi.com/journals/sv/aip/9524206/. Accessed 28 June 2016

[CR23] Lang J (1971) Result of measurements on the control of structure-borne noise from subways. In: Seventh international congress on acoustics, Budapest, pp 421–424

[CR24] Li JC, Li HB, Ma GW (2013). Assessment of underground tunnel stability to adjacent tunnel explosion. Tunn Undergr Space Technol.

[CR25] Lombaert G, Degrande G, Kogut J (2006). The experimental validation of a numerical model for the prediction of railway induced vibrations. J Sound Vib.

[CR26] Luo G, Chen JX, Zhou XJ (2015). Effects of various factors on the VIV-induced fatigue damage in the cable of submerged floating tunnel. Pol Marit Res.

[CR27] Ma M, Liu W, Qian CY, Deng GH, Li YD (2016). Study of the train-induced vibration Impact on a historic Bell Tower above two spatially overlapping metro lines. Soil Dyn Earthq Eng.

[CR29] Mata M (1971). Effects on buildings of vibrations caused by traffic. Build Sci.

[CR30] Meng ZB (2009) Analysis and assessment of the vibration responds traffic-induced of Xi’an Bell Tower, Ph.D. Thesis, Xi’an University of Architecture and Technology

[CR28] MIDAS Co. Ltd. MIDAS/NX manual. http://manual.midasuser.com/encommon/GTS%20NX/250/GTX.htm

[CR31] Real J (2014). Computational considerations of 3-D finite element method models of railway vibration prediction in ballasted tracks. J Vibroeng.

[CR32] Real T, Zamorano C, Ribes F (2015). Train-induced vibration prediction in tunnels using 2D and 3D FEM models in time domain. Tunn Undergr Space Technol.

[CR33] Rueker W (1982). Dynamic behavior of rigid foundations of arbitrary shape on a half-space. Earthq Eng Struct Dyn.

[CR34] Swiss Association of Standards (1992) Effects of vibration on construction, SN 640312, CH8008, Zurich

[CR36] Xie DW (2008) Evaluation report of vibration influence to historic and sensitive buildings along Beijing Subway Line8, Ph.D. Thesis, Beijing Jiaotong University

[CR37] Ye F, Gou CF, Sun HD (2014). Model test study on effective ratio of segment transverse bending rigidity of shield tunnel. Tunn Undergr Space Technol.

[CR38] Ye M, Cao BX, Ren YP (2015). Field measurement, analysis and protection for the vibration of an ancient ruin induced by railway. J Vibroeng.

[CR39] Yu MH, Fang DP (2006). Advances in structural mechanics of Chinese ancient architectures. Adv Mech.

[CR40] Zhao HB, Long Y, Ji C (2015). Study on the dynamic response of subway tunnel by viaduct collapsing vibration and the protective measures of reducing vibration. J Vibroeng.

